# Variations and trade-offs in leaf and culm functional traits among 77 woody bamboo species

**DOI:** 10.1186/s12870-024-05108-2

**Published:** 2024-05-10

**Authors:** Xiong Liu, Shixing Zhou, Junxi Hu, Xingcheng Zou, Liehua Tie, Ying Li, Xinglei Cui, Congde Huang, Jordi Sardans, Josep Peñuelas

**Affiliations:** 1 College of Forestry, Sichuan Agricultural University, Chengdu, 611130 China; 2 National Forestry and Grassland Administration Key Laboratory of Forest Resources Conservation and Ecological Safety on the Upper Reaches of the Yangtze River & Forestry Ecological Engineering in the Upper Reaches of the Yangtze River Key Laboratory of Sichuan Province, SICAU, Chengdu, 611130 China; 3 CREAF, Cerdanyola del Vallès, Catalonia, 08193 Spain; 4 Global Ecology Unit, CSIC, CREAF-CSIC-UAB, Bellaterra, Catalonia, 08193 Spain; 5 Institute for Forest Resources and Environment of Guizhou, Key Laboratory of Forest Cultivation in Plateau Mountain of Guizhou Province, College of Forestry, Guizhou University, Guiyang, 550025 China; 6 College of Grassland Science, Beijing Forestry University, Beijing, 100091 China

**Keywords:** Plant functional traits, Trait trade-offs, Leaf economics spectrum, Biogeochemical niche, Phylogeny, Common garden, Woody bamboo

## Abstract

**Background:**

Woody bamboos are the only diverse large perennial grasses in mesic-wet forests and are widely distributed in the understory and canopy. The functional trait variations and trade-offs in this taxon remain unclear due to woody bamboo syndromes (represented by lignified culm of composed internodes and nodes). Here, we examined the effects of heritable legacy and occurrence site climates on functional trait variations in leaf and culm across 77 woody bamboo species in a common garden. We explored the trade-offs among leaf functional traits, the connection between leaf nitrogen (N), phosphorus (P) concentrations and functional niche traits, and the correlation of functional traits between leaves and culms.

**Results:**

The Bayesian mixed models reveal that the combined effects of heritable legacy (phylogenetic distances and other evolutionary processes) and occurrence site climates accounted for 55.10–90.89% of the total variation among species for each studied trait. The standardized major axis analysis identified trade-offs among leaf functional traits in woody bamboo consistent with the global leaf economics spectrum; however, compared to non-bamboo species, the woody bamboo exhibited lower leaf mass per area but higher N, P concentrations and assimilation, dark respiration rates. The canonical correlation analysis demonstrated a positive correlation (ρ = 0.57, *P*-value < 0.001) between leaf N, P concentrations and morphophysiology traits. The phylogenetic principal components and trait network analyses indicated that leaf and culm traits were clustered separately, with leaf assimilation and respiration rates associated with culm ground diameter.

**Conclusion:**

Our study confirms the applicability of the leaf economics spectrum and the biogeochemical niche in woody bamboo taxa, improves the understanding of woody bamboo leaf and culm functional trait variations and trade-offs, and broadens the taxonomic units considered in plant functional trait studies, which contributes to our comprehensive understanding of terrestrial forest ecosystems.

**Supplementary Information:**

The online version contains supplementary material available at 10.1186/s12870-024-05108-2.

## Introduction

Woody bamboos, the only diversified large perennial grasses in forests, diverged from herbaceous ancestors approximately 42 million years ago, rapidly radiating into the understory and canopy of mesic-wet forests worldwide [1–3]. They occupy vital ecological niches in forest ecosystems and influence forest regeneration, community composition and dynamics [4–8]. Unlike herbaceous or common woody, woody bamboo syndromes include strongly lignified culms, specialized culms leaves, gregarious and usually monocarpic flowering [2, 9], which may drive the specificity of interspecific functional trait variations and trait relationships in this taxon. However, interspecific variations and trait trade-offs in functional traits of woody bamboo have remained underexplored, limiting our understanding of ecological adaptive strategies of woody bamboos and the comprehensive views of forest ecosystems.

The leaf economic spectrum (LES) of leaf mass per area, leaf lifespan, assimilation rate, dark respiration rate, nitrogen (N) concentration and phosphorus (P) concentration trade-offs among species based on the global plant trait network (GLOPNET, 2548 species) reflects the strategic gradient of plant carbon gain and life history from slow (conservation) to fast (acquisition) [10]. However, the GLOPNET dataset includes only one woody bamboo species (*Bambusa bambos*), so it is unknown whether similar LES tradeoffs exist in woody bamboo taxa. Indeed, the previous empirical studies support the distinctions in leaf functional traits between woody bamboos and other species. For example, higher leaf N concentration and lower leaf mass per area for woody bamboos than for trees and lianas have been reported in the Amazon rainforest [11, 12]. In addition, woody bamboos were believed to have evolved in forests with higher competition for light [13] and consistently currently possess higher photosynthetic efficiency than most grasses despite using the C_3_ photosynthetic pathway [14]. Regarding the trade-offs in leaf traits, although positive correlations between N and P concentrations and assimilation rates in the leaves of five bamboo species have been reported [15], similar to the global LES [10], another study showed that the maximum photosynthesis rate of *Sasa veitchii* was uncorrelated with leaf mass per area and area-based leaf N concentration [16]. These cases clearly show that woody bamboo functional traits and trait trade-offs may differ from those of non-bamboo species, but empirical evidence is still lacking in larger woody bamboo datasets.

Woody bamboo lignified culms have no secondary cambium and consist of hollow internodes and nodes [2, 17], a structure unique to this taxon that may alter culm-leaf functional trait correlations. For woody bamboo, internode length of culm represents possible trade-offs mediated by mechanical pressure caused by the rapid growth [18], while the ground diameter is closely related to the whole plant size and bamboo biomass [19]. Studies on global plant form and function suggest that the LES and the traits reflecting plant sizes belong to two dimensions [20], indicating the independence of stems and leaves in evolutionary history, supported by several empirical studies [21, 22]. However, some studies claimed that the relevant economic traits in an individual should follow the whole-plant “fast-slow” coordination mechanism [23], which was confirmed in some hydraulic traits [24]. Therefore, although the relative independence and orthogonality functional trait associations between stem (or culm) and leaf have been reported in non-bamboo grasses [25], trees and shrubs [20], it is still unclear whether this rule applies to woody bamboo.

Functional traits variations and trade-offs are determined by genetic evolution and natural selection [26, 27], and evolve along phylogenetic branches [28]. As closely related species have a more common evolutionary history than distantly related species, they are often more consistent in functional traits, a pattern known as phylogenetic niche conservatism [29]. However, species may change certain traits quickly to adapt to new habitat climates or compete with other coexisting species, leading to variation within the phylogenetic spectrum, e.g., adaptive radiation [28, 30]. For instance, the recently proposed Biogeochemical Niche (BN) hypothesis predicts that broad interspecific variation in foliar elemental composition (elementome) would be strongly controlled by phylogeny (common ancestry and phylogenetic distances), and regulated by recent convergent and divergent evolutionary processes (e.g., adaptive radiation and epigenetic evolution), as well as occurrence abiotic factors [31], and has been validated in trees [32, 33]. Additionally, a recent study has found significant correlations between the foliar elementome and the morphological functional traits in leaves, except for LES trade-offs, possibly indicating that functional-morphological niches and species-specific biogeochemical use are likely to have co-evolved [32]. Unfortunately, woody bamboos were virtually absent from the relevant studies.

**Fig. 1:**
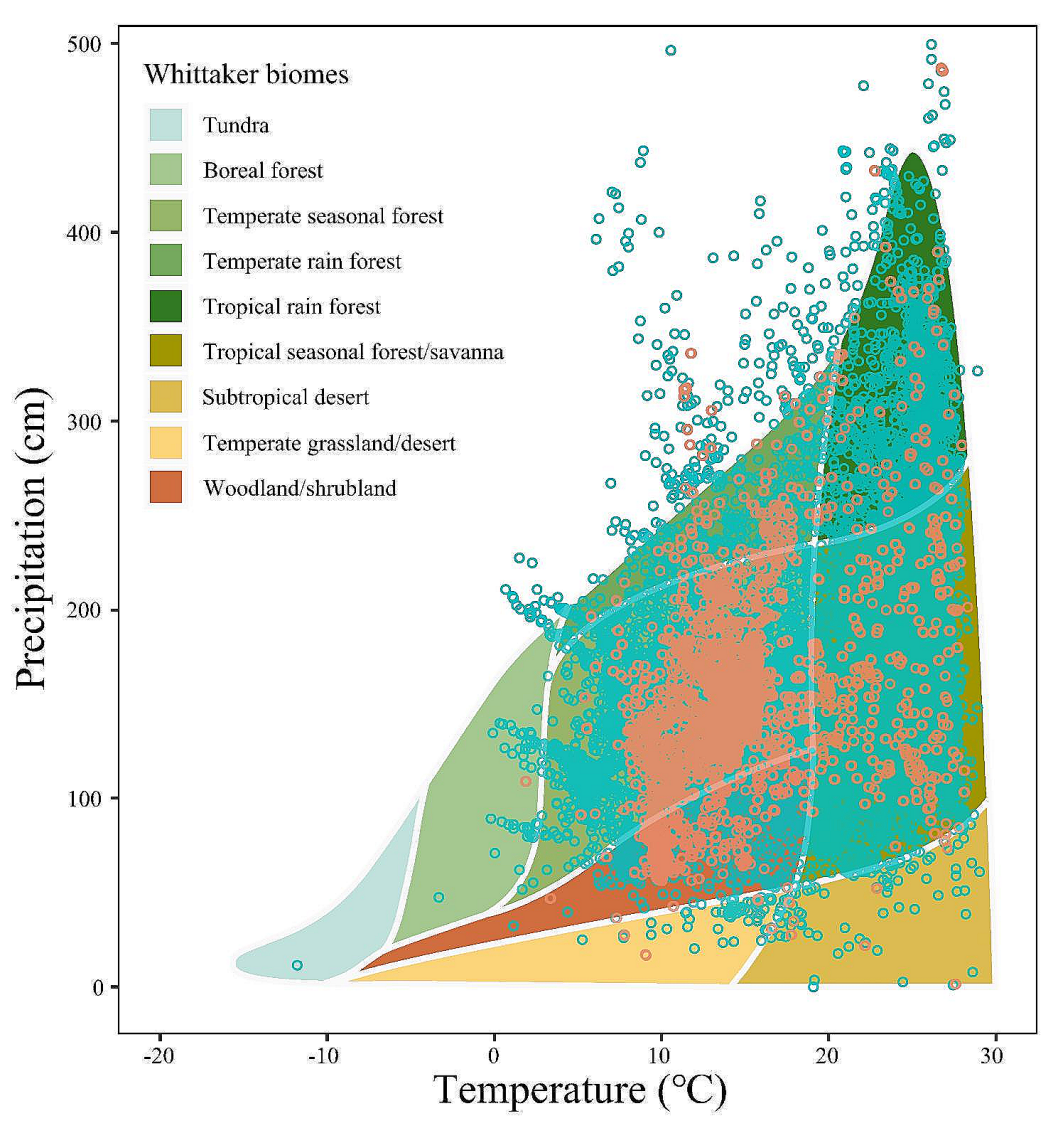
Temperature and precipitation values from WorldClim based on the woody bamboo occurrence records in GBIF. The cyan circles represent the climate observations of the global woody bamboo occurrence sites (*n* = 1017), and the orange-red circles represent the climate observations of this study woody bamboo occurrence sites (*n* = 54), more than one occurrence record for each species. All woody bamboo species occurrence sites were from GBIF (www.gbif.org), and temperature and precipitation observations were from WorldClim (www.worldclim.org). Occurrence sites from the greenhouse were also included in the figure. Biome boundaries were approximate.

To address these gaps, we measured the LES traits (leaf mass per area, N and P concentrations, maximum assimilation rate and dark respiratory rate) and the culm traits (ground diameter and internode length) of 77 woody bamboo species from 21 genera in a common garden. These woody bamboos, native to seven Asian countries, occur across tropical to temperate climate zones, ranging from lowland forests to alpine forests (Table S1), covering almost all habitat types of woody bamboo worldwide (Fig. 1). We quantified the functional traits and their variations of these woody bamboos and analyzed the effects of heritable legacy (phylogenetic and short-term evolutionary processes) and occurrence site climates on trait variations. Additionally, we analyzed the trade-offs for leaf functional traits and compared them with the global LES. Furthermore, we examined the trait correlations between leaves and culms. Specifically, our study aimed to answer the following questions among woody bamboo species: (1) To what extent do heritable legacy and occurrence site climates affect the functional trait variations among woody bamboo? (2) Is there a LES across woody bamboo species, and if so, does it align with the global LES pattern? (3) Is the BN hypothesis consistent across woody bamboo species and N and P concentrations related to the morphophysiology functional niche? (4) Are the functional traits of culms and leaves in woody bamboo correlated? Our study thus aims to enhance the understanding of the functional traits of woody bamboo and expand the taxa of the global plant trait study.

## Materials and methods

### Study site

The study site is located at the International Bamboo Germplasm Resource Bank of Wangjiang Tower Park (Chengdu, China) (30°37′45.48″N, 104°05′33.78″E) and an elevation of 485 m asl, where estimated mean annual temperature is 15.5 ℃ and mean annual precipitation is 1024 mm (Chengdu Meteorological Bureau) under a subtropical humid monsoon climate. The common garden comprised 77 woody bamboo species from 21 genera (Table S1) and planted from June 2014 to June 2016. We unified synonyms from the original references to unique and consistently accepted species names following the WFO Plant List nomination system (wfoplantlist.org/plant-list). We queried the occurrence records of all woody bamboo species worldwide and the present study through GBIF (www.gbif.org) and then queried the temperatures and precipitations at the occurrence sites at WorldClim (www.worldclim.org), which confirmed that the 77 woody bamboo species in the present study were broadly representative (Fig. 1). The post-planting management of these bamboos was irrigation from 07:00 to 08:00 a.m. following two days of no rain. All the woody bamboos were well established and had successfully propagated by shoots for three consecutive years; there were no pest or disease outbreaks during the experiment.

### Field experiment and functional traits measurement

We measured nine functional traits that represent the two axes of plant trait space [10, 20], including ground diameter (GD) and maximum internode length (ITL) as measures of the whole plant and representative internode [18], respectively, and leaf mass per area (LMA), maximum assimilation rate based on area (A_area_) and mass (A_mass_), dark respiration rate based on area (Rd_area_) and mass (Rd_mass_), leaf nitrogen concentration (N), and leaf phosphorus concentration (P) as measures of LES traits (Table 1), following the standard protocols [34], as detailed below.

We selected five 2–3 years-old non-clonal individuals of average height for each species and measured the GD and ITL in June 2021 using vernier calipers (0.1 cm, DL91150, Deli Group Co. LTD, Ningbo, China) and tapeline (0.1 cm, DL91150, Deli Group Co. LTD, Ningbo, China); if the culms were irregular cylinders, we derived the mean GD from measurements of maximum and minimum GD.

We randomly selected three individuals from the above five ones, per species selected three fully expanded leaves for the A_area_ and Rd_area_ measurement from 09:00 to 12:00 a.m. between July and August 2021 using a portable photosynthesis system (LI-COR 6800, LI-COR Corporate, Nebraska, USA) under cloudless conditions; ambient air in the leaf chamber was maintained at 20°C, relative humidity was 60%, and CO_2_ concentration of the incoming air was 400 µmol L^− 1^. We estimated the photosynthetic light-response curve for each leaf using photosynthetically active radiation (PAR) levels of 0, 25, 50, 75, 100, 200, 400, 600, 900, 1200, 1500, and 1800 µmol m^− 2^ s^− 1^ provided by LI-COR 6800 [35]. The A_area_ and Rd_area_ were calculated using the Hyperbolic Modified Model in Photosynthesis Calculation 4.1.1 (School of Mathematics and Physics, Jinggangshan University, Ji’ an, Jiangxi, China) by importing the light intensity (I) and the corresponding net photosynthetic rate (P_n_) from the above light-response curve [36, 37]. We then calculated the A_mass_ and Rd_mass_ by the following formulas:

1 $${A}_{mass}=\frac{{A}_{area}\times 1000}{LMA}$$

Here the A_area_ represent area-based maximum assimilation rate (µmol m^− 2^ s^− 1^); the LMA represent leaf mass per area (g m^− 2^); the A_mass_ represent mass-based maximum assimilation rate (nmol g^− 1^ s^− 1^).

2 $${Rd}_{mass}=\frac{{Rd}_{area}\times 1000}{LMA}$$

Here the Rd_area_ represent area-based dark respiration rate (µmol m^− 2^ s^− 1^); the LMA represent leaf mass per area (g m^− 2^); the Rd_mass_ represent mass-based dark respiration rate (nmol g^− 1^ s^− 1^).

Then, we collected 100 g sunny leaves from the upper, mid, and lower parts with similar proportions of these individuals for measurement of LMA, N, and P in July 2021; they were mixed to form a single composite sample per individual and immediately taken to the laboratory. For the measurement of LMA, 30 randomly selected, fully expanded leaves were scanned at 300 dpi (BenQ-5560, BenQ Corporation, Shanghai, China) and the leaf areas were subsequently measured using IMAGE J 1.8.0 (Wayne Rasband-National Institute of Health, Bethesda, MD, USA); then, the scanned leaves were oven-dried at 60 °C and weighed, and LMA was calculated as leaf dry mass/fresh area. The remaining collected leaves were dried at 60 °C for 72 h, then crushed using a pulverizer (CS-700, Wuyi Haina Electric Appliance Co., LTD, Jinhua China) and passed through a 100-mesh sieve prior to leaf N and P concentrations (%) determination using the Hanon K9840 auto-Kjeldahl analyzer (Jinan Hanon Instruments Co., Ltd., Jinan, China) and the molybdenum blue colorimetric method (following digestion in a H_2_SO_4_ and H_2_O_2_ solution) [34], respectively.

### Temperature and precipitation data of woody bamboo occurrence sites

To assess the effects of temperature and precipitation of woody bamboo occurrence sites on functional trait variations of woody bamboo, we extracted species names of 1592 woody bamboos through the RGB Kew: Grassbase (www.kew.org/data/grassbase) and retained 1492 species after correction in The WFO Plant List (wfoplantlist.org). The occurrence records of 1492 woody bamboos were extracted in GBIF (www.gbif.org) with rgbif package [38], of which 1017 species were available, containing 54 bamboo species in this study. We extracted temperature and precipitation for all distribution sites of 1017 species by occurrence records in WorldClim (www.worldclim.org) (Fig. 1). Since occurrence records for greenhouses were also included, to eliminate errors caused by extreme values, we chose to use median rather than mean values in this study to assess the effects of climate and precipitation on functional trait variations in woody bamboo.

### GLOPNET dataset

To compare the LES of woody bamboos with non-bamboo species, we used the GLOPNET dataset from which LES was proposed [10]. We extracted the values of the same five leaf traits (LMA, N, P, A_mass_, and Rd_mass_) of non woody bamboo species (2547 species, excluding only one woody bamboo species, *Bambusa bambos*) from the GLOPNET dataset [10] for a consistent analysis of trait-trait trade-offs. We expressed all leaf economics traits on a mass basis in this analysis.

### Statistical analysis

#### Effect of heritable legacy and climate on functional traits

We utilized the S.PhyloMaker() function from the phytools package in R [39] to retrieve a phylogenetic tree compassing the species under investigation in this study. This tree was derived from the PhytoPhylo species-level phylogenetic megatree, constructed using gene sequences sourced from GenBank for land plants [40, 41]. Previous studies have demonstrated that phylogenies generated by S.PhyloMaker() using the PhytoPhylo mega phylogeny as a scaffold exhibit nearly equivalent accuracy to the phylogenies resolved at the species level when quantifying quantify phylogenetic properties (e.g., phylogenetic diversity and phylogenetic relatedness) of biological assemblages [40, 42].

We tested the effects of heritable legacy and bamboo occurrence site climates on the functional trait variations using Bayesian phylogenetic mixed models and the MCMCglmm package in R [43]. Heritable legacy effects, represented by phylogeny and species, were incorporated as random factors in our analysis. The phylogenetic term addressed variability resulting from shared ancestry, capturing the influence of the common evolutionary history of each set of samples of the same species and separating them from samples of other species by the non-shared times derived from phylogenetic distances. On the other hand, the species term accounted for species-specific traits that are independent and not explained by phylogenetic distances. These traits are influenced by more recent evolutionary phenomena not captured by long-time phylogenetic distances, including convergent and divergent evolutionary processes, along with epigenetic evolution. The combined inclusion of both random factors effectively accounted for the variance explained by heritability [33].

#### Bivariate relationships of leaf functional traits

The slopes and associated coefficients of determination (R^2^) for species trait pairs for LMA, N, P, A_mass_, and Rd_mass_ were calculated using a standardized major axis (SMA) analysis [44]; the slopes and R^2^ were then compared with trait-trait relationships for non-bamboo plants in the GLOPNET dataset [10]. The SMA was conducted with the sma() and ma() function in the mart package [44].

#### Relationship of leaf N and P concentrations and functional niche

We examined the relationship between leaf elemental, represented by foliar N and P concentrations, and the functional niche traits, represented by LMA, A_mass_, and Rd_mass_, which was analyzed using the canonical correlation analysis (CCA) from the CCA package [45]. The CCA aims to analyze the correlation of paired linear combinations between two groups variables, while the linear combinations within the group are uncorrelated, and preserves as much information as possible about the original variables [45]. The root scores were extracted from the CCA results and correlation analyses were performed with the CCP package, which provides functions to test the statistical significance of typical correlation coefficients [46]. The significance of this correlation was confirmed by the Wilks’ Lambda test of p.asym() function in the CCP package [46].

#### Correlation of bamboo culm and leaf functional traits

Given the Bayesian phylogeny results showed the strong effects of heritable legacy on functional trait variations in woody bamboo, thus we used the phylogenetic principal components analysis (pPCA) to test for multivariate covariant relationships among the leaf and culm functional traits. The pPCA was carried out using the phyl.pca() function in R package phytools [47].

Plant trait networks (PTNs) analysis was used to test for organ-level associations between bamboo leaf and culm functional traits (correlation coefficient = 0.5, *P*-value < 0.05). The PTNs is an effective method to explore the complex relationship between multiple plant traits [48]. In PTNs, the degree is the sum of edges that connect focal node traits to other nodes, and the traits that have a higher degree favor the efficient use and acquisition of resources [23], which were delineated as ‘hub traits’ in PTNs; and the betweenness is the number of shortest paths passing through a focal node trait, the traits with a higher betweenness serve as brokers (i.e. traits with high betweenness likely coordinate several subnetworks) [49]. The PTNs was visualized using the igraph package [50]. All statistical analyses were run in R 4.2.0 [39].

## Results

### Functional trait variations

Functional traits showed high variations across the 77 woody bamboo species (Table 1), with more than ten-fold ranges in values shown for GD, ITL and Rd_area_, and more than three-fold ranges in values shown for LMA, N, P and A_area_. GD had the highest coefficient of variation for 86.63%, while N had the lowest coefficient of variation for 20.96%.

**Table 1:** Descriptive statistics of the functional traits across 77 woody bamboo species

Organs	Functional traits	Abbreviations	Units	Mean	Range	CV (%)	
Culm	Ground diameter	GD	cm	3.81	0.20–16.00	86.83	
	Maximum internode length	ITL	cm	28.55	5.00–56.00	40.44	
Leaf	Leaf mass per area	LMA	g m^− 2^	37.22	12.65–77.69	35.89	
	Leaf N concentration	N	%	1.84	0.93–3.13	20.96	
	Leaf P concentration	P	%	0.12	0.07–0.21	24.16	
	Maximum assimilation rate	A_area_	µmol m^− 2^ s^− 1^	10.95	3.43–22.28	41.99	
	Dark respiration rate	Rd_area_	µmol m^− 2^ s^− 1^	1.34	0.36–4.38	60.93	

CV: coefficient of variation (100 × standard deviation/mean)

### Heritable legacy and climate effects on functional traits

**Fig. 2:**
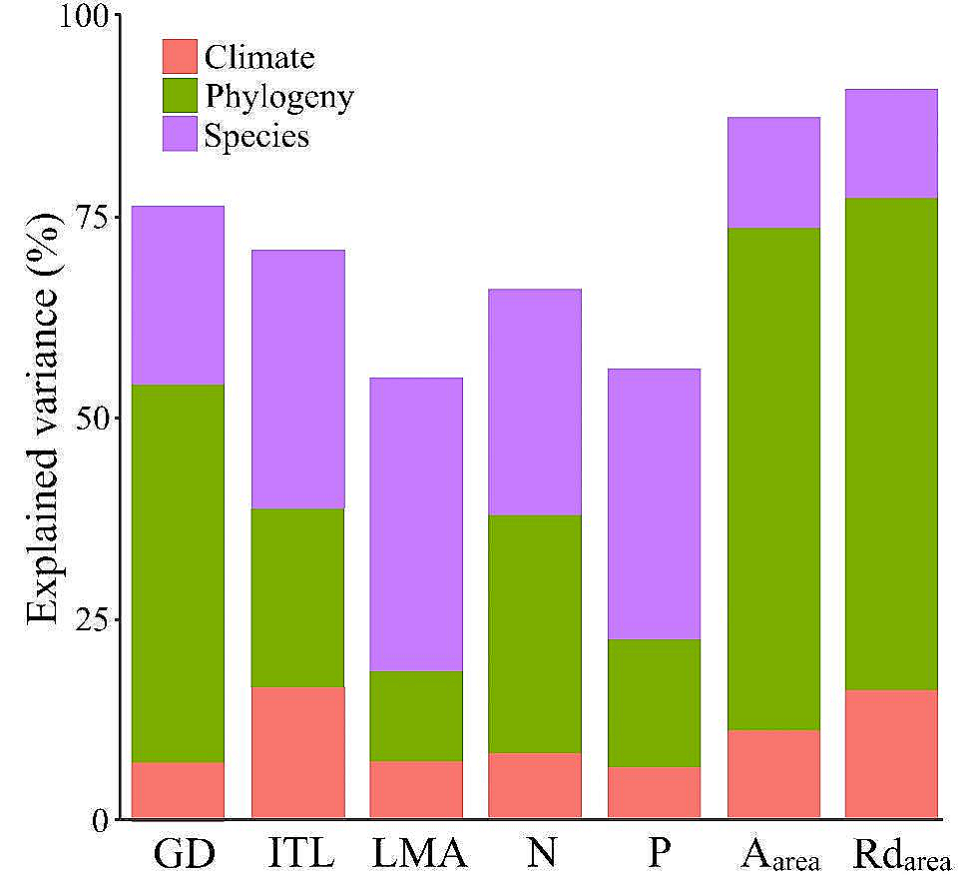
Percentage of trait variations explained by Bayesian phylogenetic mixed models. Climate: temperature and precipitation. Refer to Table 1for trait abbreviations

The Bayesian phylogenetic mixed models showed that heritable legacy, together with the occurrence site climates, explained between 55.10% and 90.89% (mean 71.87%) of the variation in functional traits of woody bamboo (Fig. 2, Table S2). The variance decomposition results revealed that temperature and precipitation collectively accounted for 7.31–16.69% (mean 10.63%) of the total trait variations. Phylogeny contributed to 11.18–62.38% (mean 35.57%), while species explained 13.54–36.41% (mean 25.66%) of the total trait variations (Fig. 2, Table S2). Heritable legacy, encompassing both phylogeny and species, elucidated 47.59–76.10% (mean 61.23%) of the functional trait variations in woody bamboo (Fig. 2, Table S2).

### Bivariate relationships of leaf functional traits

**Fig. 3:**
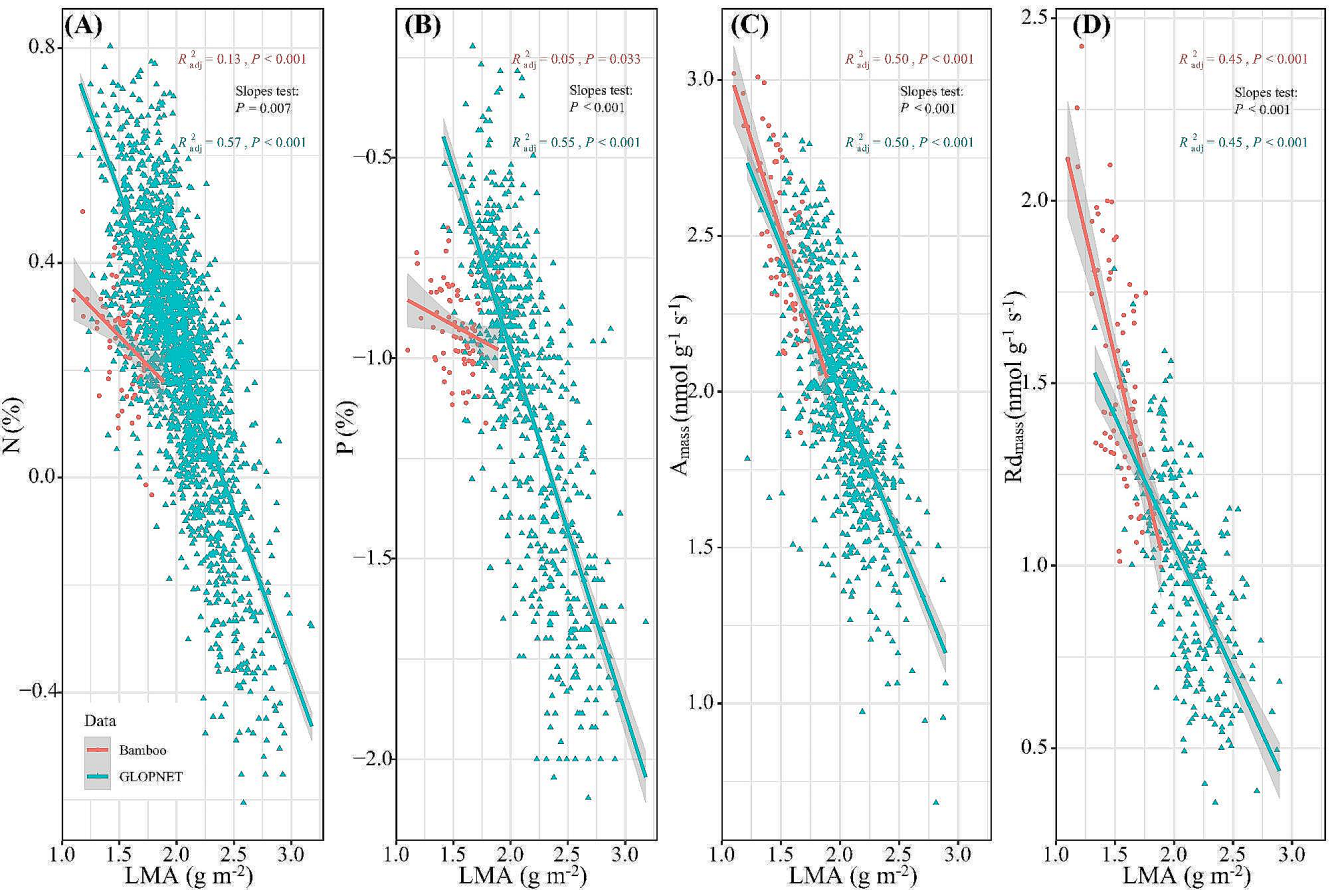
The bivariate trait relationships of leaf N, P, A_mass_, Rd_mass_ with LMA for woody bamboo and non-bamboo species. Solid line is the fitted relationship (*P*-value < 0.05) with 95% CIs (shaded area), and comparison in slopes tests for differences between trait relationships in bamboo and non-bamboo species (*P*-value < 0.05). Non-bamboo species are from GLOPNET dataset [10]. Refer to Table 1 for trait abbreviations

**Fig. 4:**
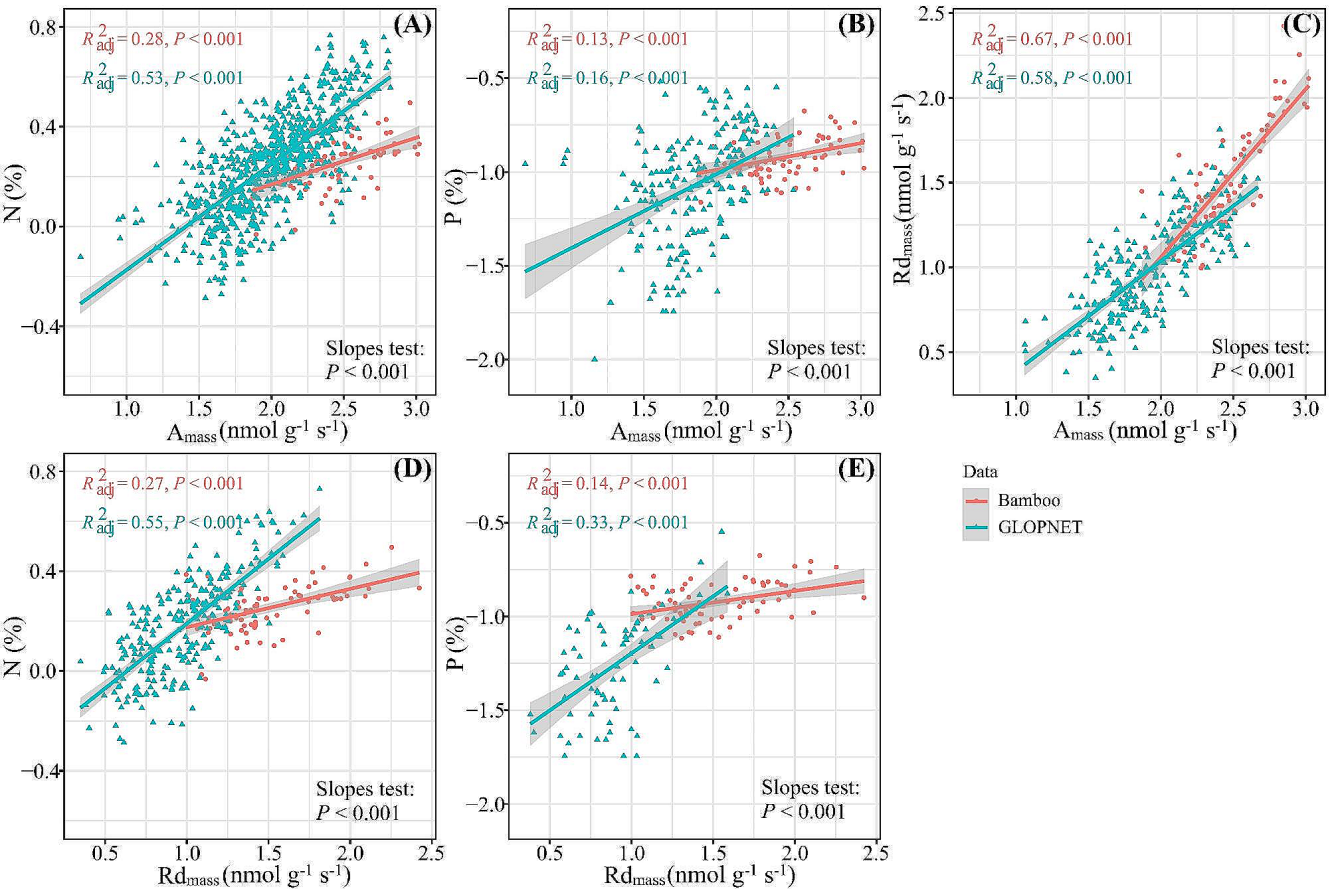
The bivariate trait relationships between leaf N, P, A_mass_, and Rd_mass_ for woody bamboo and non-bamboo species. Solid line is the fitted relationship (*P*-value < 0.05) with 95% CIs (shaded area), and comparison in slopes tests for differences between trait relationships in bamboo and non-bamboo species (*P*-value < 0.05). Non-bamboo species are from GLOPNET dataset [10]. Refer to Table 1 for trait abbreviations

The directions of the bivariate relationship between woody bamboo leaf traits were consistent with the global leaf economics spectrum, with negative relations between LMA and leaf traits (Fig. 3A-D) and positive relations between leaf traits and A_mass_ and Rd_mass_ (Fig. 4A-E). The relationships between LMA and N and P concentrations (Fig. 3A-B), between A_mass_ and N and P concentrations (Fig. 4A-B), and between Rd_mass_ and N and P concentrations (Fig. 4D-E) showed flatter slopes for bamboo than non-bamboo species, while the relationships between LMA and A_mass_ and Rd_mass_ (Fig. 3C-D) and between A_mass_ and Rd_mass_ (Fig. 4C) showed steeper slopes for bamboo than non-bamboo species (*P*-values < 0.05).

### Relationship of leaf N and P concentrations and functional niche

**Fig. 5:**
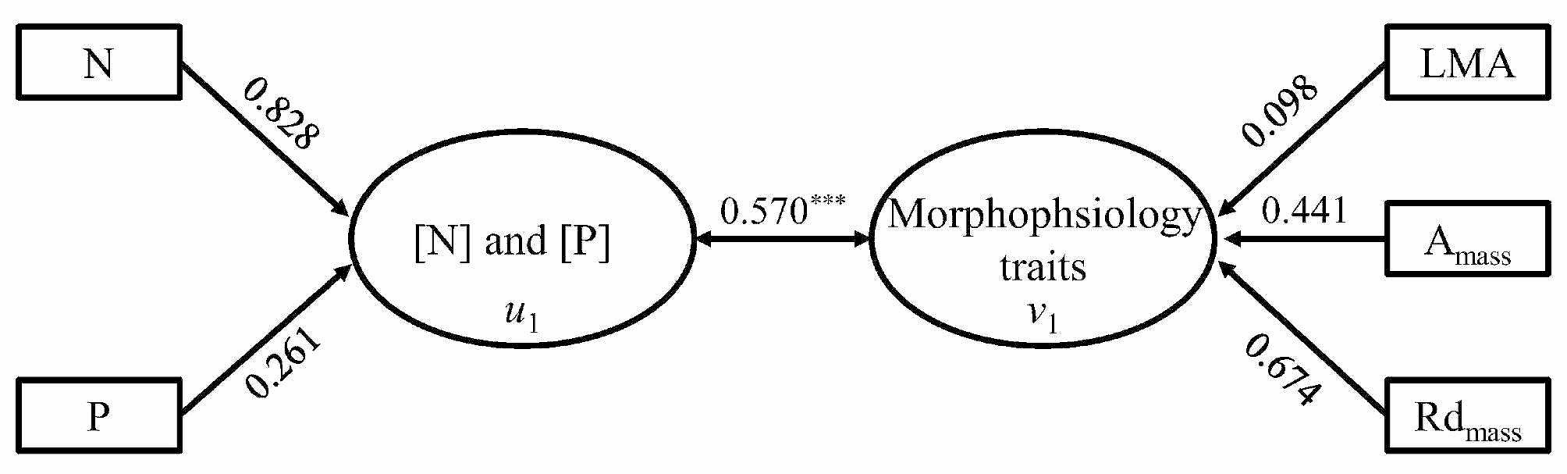
The canonical correlation analysis of leaf elemental N and P concentrations and morphophysiology traits across 77 woody bamboo species. *u*1: root 1 scores of foliar N and P concentrations; *v*1: root 1 scores of morphophysiology traits. ^***^P-value < 0.001. Refer to Table 1 for trait abbreviations

The canonical correlation analysis revealed that the root 1 scores between leaf N and P concentrations and morphophysiology traits exhibited a significantly positive correlation with a correlation coefficient of 0.570 (*P*-value < 0.001, Fig. 5, Fig. S1A, Table S3). In the elemental composition, the coefficients for N and P were 0.828 and 0.261, respectively. Regarding morphophysiology traits, the coefficients for LMA, A_mass_, and Rd_mass_ were 0.098, 0.441, and 0.674, respectively (Fig. 5).

### Association between culm and leaf functional traits

**Fig. 6:**
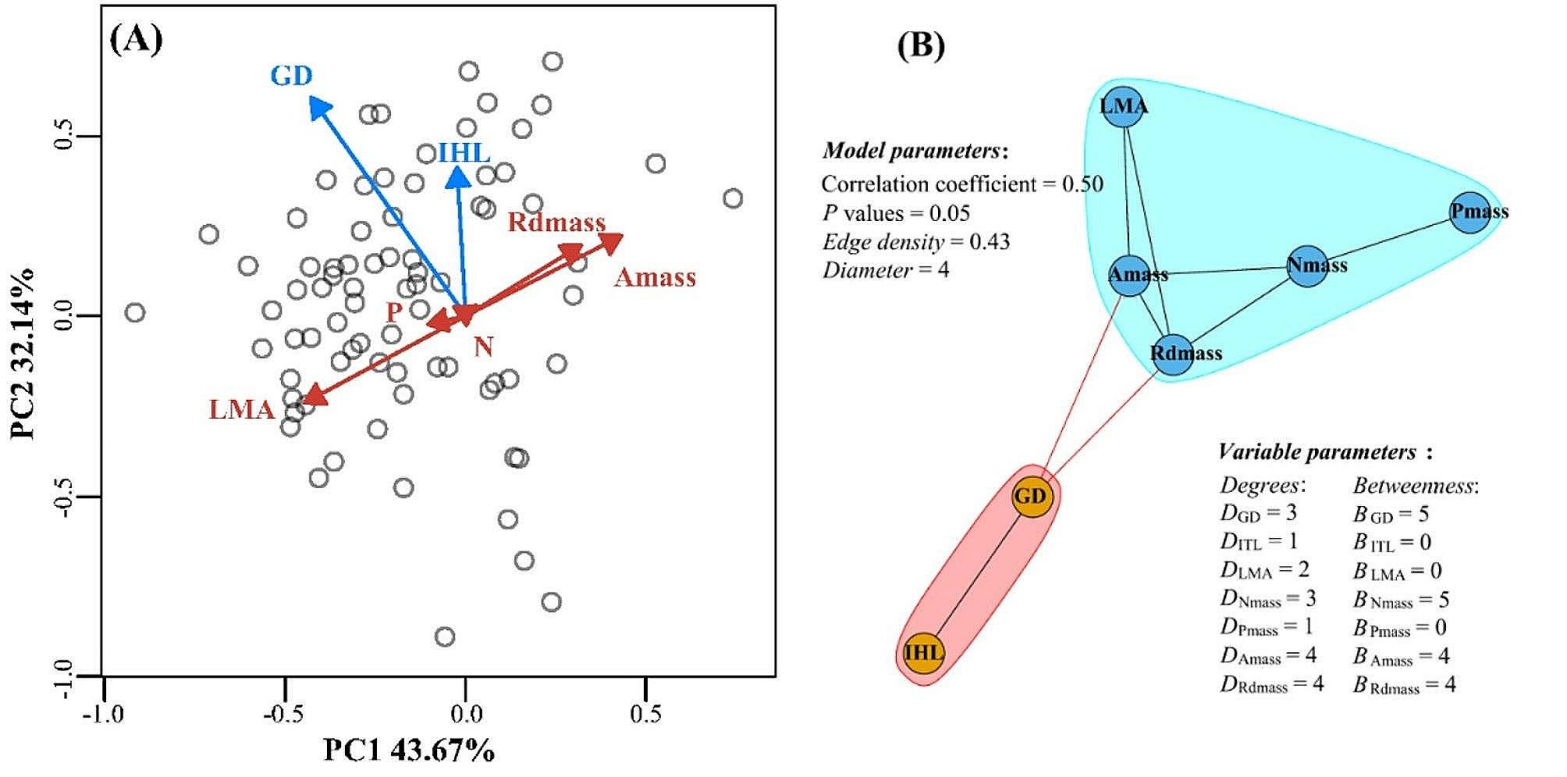
The pPCA **(A)** and PTNs analysis **(B)** results of leaf and culm traits for 77 woody bamboo species. The light-blue arrows and circles represent leaf traits and the dark-red arrows and light-yellow circles represent culm traits. The Degree (*D*) represents the sum of edges that connect the focal node traits to other nodes and the Betweenness (*B*) represents the number of shortest paths going through a focal node trait. Refer to Table 1 for trait abbreviations

The first two axes of pPCA analysis explained for 75.81% of the variation in bamboo functional traits, with leaf traits mainly weighted in the first axis and culm traits mainly weighted in the second axis, which tended to be orthogonal (Fig. 6A). The PTNs analysis showed that the leaf and culm functional traits were clustered separately and connected by GD, A_mass_ and Rd_mass_ (Fig. 6B). The degrees of functional trait ranged from 1 to 4, with A_mass_ and Rd_mass_ characterized by the greatest degrees of 4. The betweenness of functional traits ranged from 0 to 5, with GD characterized by the greatest betweenness of 5 in the culm and N of 5 in the leaf, respectively, and A_mass_ and Rd_mass_ have the second betweenness of 4 in the leaf (Fig. 6B).

## Discussion

### Heritable legacy and climate effects on functional trait variations

We found high interspecific variations in leaf and culm functional traits among the 77 woody bamboo species in the common garden (Table 1). The Bayesian phylogenetic mixed models showed that most of the trait variations (55.10–90.89%) could be explained by heritable legacy and climate factors (Fig. 2). Among them, heritable legacy (including phylogeny and species) accounted for 47.59–76.10% of the total variations (Fig. 2, Table S2), and the effect of occurrence site climates accounted for 6.75–16.69% (Fig. 2, Table S2), similar to the results of previous studies on elemental stoichiometry in leaves [33]. On the one hand, since the Paleogene period, the woody bamboo clade has undergone diversification into a wide variety of morphologically distinct species [51, 52], with evolutionary history and genetics contributing to extensive interspecific variation in functional traits [53, 54]. For instance, the 77 woody bamboo species from 21 genera in this study including lianas (e.g. *Drepanostachyum stoloniforme*), shrubby (e.g. *Fargesia stenoclada*) and arborescent (e.g. *Dendrocalamus sinicus*) (Table S1), thus resulted in high interspecific coefficients of variation in their ITL and GD (86.83% and 40.44% respectively). On the other hand, woody bamboos have rapidly radiated into the mesic-wet forests worldwide, and natural selection and adaptive radiation driven by diverse habitats have resulted in high interspecific variation in functional traits [55]. For example, the 77 woody bamboo species in our study currently occur in three climatic zones, from temperate (e.g. *Semiarundinaria*), subtropical (e.g. *Phyllostachys*) to tropical (e.g. *Dendrocalamus*), and altitudes from 200 m (e.g. *Gigantochloa*) to 3600 m (e.g. *Fargesia*) (Table S1). Although short-term (compared to evolutionary history) common garden planting may alter the range of functional trait intraspecific variations, the long-term effects of climate in species occurrence sites in evolutionary history on functional trait variation between species should not be underestimated [56], just like the effects of temperature and precipitation (6.75–16.69%) in our Bayesian mixed models.

Among the functional traits we tested, the GD, A_area_, and Rd_area_ exhibited greater phylogenetic weights (46.90–62.38%) and lower species (other evolutionary variables not related to phylogenetic distances) weights (13.54–22.22%). This implies that these traits have been relatively stable during evolution and are more influenced by the distinct evolutionary history among species [33]. In contrast, the traits like ITL, LMA, N, and P showed higher species-specific weights (28.02–36.41%) and lower phylogenetic effects (11.18–29.55%). This suggests that these traits may have undergone significant short-term adaptive radiations to adapt to different habitats [28, 57]. Furthermore, the observed significant effects in the elemental composition variations (N and P) among species due to heritable legacy align with the Biogeochemical Niche (BN) hypothesis [31–33]. To our best knowledge, this is the first time BN hypothesis has been tested among multiple woody bamboo species. Notably, our study did not include soil conditions and forest community structure, which could account for residuals in the Bayesian mixed models [33]. In summary, the interspecific variation in functional traits among 77 woody bamboo species is co-regulated by phylogeny, species and occurrence site climates, with the heritable legacy playing a dominant role and the climate acting as a relative moderating factor. This interaction shapes the functional traits’ high interspecific variations, phylogenetic conservatism and biogeochemical ecological niches in woody bamboos.

### Bivariate relationships of leaf functional traits

We confirmed the LES among 77 woody bamboo species, which clustered at the acquisitive end of the global leaf economics spectrum, characterized by higher levels of N, P, Rd_mass_, and A_mass_ and lower LMA (Figs. 3 and 4). The woody bamboo LES was driven by the unique resource demand strategies and the typical habitat climates. Approximately 42 million years ago, woody bamboo diverged from grasses but retained the ecological strategies of fast growth and hollow culm (woody bamboo syndrome) [51] and evolved efficient reproductive strategies (numerous bamboo shoots annually) [18], which required support from high carbon and nutrient demands [58], thereby promoting the formation the LES of the fast investment-return type. Furthermore, the woody bamboos are predominantly distributed in the warm, humid temperate to tropical forests [59, 60], where adequate water supply and suitable temperature conditions accelerate physiological and metabolic rates, facilitating nutrient turnover [59] and driving the clustering of the woody bamboo LES at the acquisitive end of the global LES.

We observed two notable differences in the slopes of tradeoffs in leaf economics traits between woody bamboo and the non-bamboo species. Firstly, the woody bamboo LMA, A_mass_, and Rd_mass_ were more responsive to changes in N and P concentrations. Secondly, the woody bamboo LMA was less responsive to changes in A_mass_ and Rd_mass_, and Rd_mass_ to A_mass_. These results indicate that woody bamboos have higher nutrient use efficiency, lower leaf-building costs and faster metabolic rates than non-bamboo species in terms of carbon acquisition and utilization strategies, similar to the results of previous empirical studies [14, 61, 62]. These differences in the ecological adaptive strategies can be explained by unique habitat conditions and syndromes of woody bamboo taxon.

On the one hand, the woody bamboos mostly distribute in forest-dense formations [63], where limited light resources may drive bamboos to invest leaf nutrients in increasing leaf area to intercept more light, and small changes in N and P can dramatically alter leaf morphologic traits, thus driving a more responsive leaf nutrient concentration trade-off with LMA (Fig. 3). On the other hand, studies have found that the underground roots/rhizomes biomass allocation ratio of woody bamboo (*Phyllostachys edulis*) (44 ± 0.13%) was more than twice that of trees (22 ± 0.08%) due to syndrome [64], and a higher leaf respiration rate is required to maintain the metabolic activities of the underground rhizome system. We guess that the large amounts of carbon assimilated by photosynthesis in woody bamboo leaves are used to construct rhizome systems and result in low leaf LMA, whereas rhizome metabolism and bamboo shoot development increase nutrient demand and leaf respiration rates drive the specificity of the woody bamboo leaf economics spectrum [65], but this needs to be verified experimentally.

Additionally, our study suggests that the leaf functional traits of woody bamboo expand the GLOPNET trait space due to the significantly lower LMA of woody bamboo (37.22 g m^− 2^) compared to non-bamboo species in GLOPNET (154.78 g m^− 2^), resulting in higher A_mass_ and Rd_mass_. This underscores the necessity of incorporating woody bamboo’s functional traits into the global plant trait space.

In summary, our findings provide evidence for the LES among 77 woody bamboo species, with trade-off strategies between leaf functional traits conforming to the global model, supporting the generality of the LES in closely related species (Bambusoideae). The leaf functional traits of the woody bamboo cluster at the acquisition end of the global LES represent that woody bamboo is a fast investment-return species. The differences in the slope of the LES trait trade-offs between woody bamboo and non-bamboo species reflect the specificity of ecological adaptation strategies driven by woody bamboo syndromes.

### Relationship of leaf N and P concentrations and functional niche

We observed the significant associations between leaf N and P concentrations and morphophysiological traits (LMA, A_mass_, and Rd_mass_) across 77 woody bamboo species (Fig. 5). This finding aligns with a prior study involving 60 coexisting tree species in a tropical rainforest [32]. In our study, the N concentration was the prominent contributor to leaf elemental composition, and the A_mass_ and Rd_mass_ were the prominent contributors to morphophysiological traits (Fig. 5), suggesting that they played more important roles in determining the functional niche and ecological strategy of woody bamboo. The association of elemental composition and morphophysiological traits corresponds to established principles in plant physiology, where N plays a crucial role in key photosynthetic and respiratory processes, including photosynthetic nitrogen use efficiency and the N and P requirements for building Rubisco enzymes [66, 67]. Likewise, the relationship between woody bamboo leaf N and P concentrations and morphophysiological traits aligns with the trade-off strategy observed in the global LES. Specifically, species with high N concentration tend to exhibit elevated levels of photosynthetic performance (indicated by high A_mass_ and Rd_mass_) [10].

Our study directly confirms the association between woody bamboo leaf elemental composition, the evolutionary heritage of the species, and morphophysiological traits. This consistency with the observed in some previous studies on the relationship between leaf elemental composition and species function and ecological strategies [32] constitute a new clue on the consistence of the BN as a tool to can identify species niche by their elemental composition. Both leaf elemental composition and morphophysiological traits were subject to regulation by heritable legacy and climates [68, 69], diverging and associating within specific evolutionary histories and habitats. This dynamic interaction creates niche conservatism [29, 32] and biogeochemical niches [31, 32]. Ultimately, these niches sustain species’ biological processes and contribute to the formation of unique ecological adaptive strategies.

### Association between culm and leaf functional traits

The weak correlation between woody bamboo culm and leaf functional traits (Fig. 6A) and clustering of these traits by organ indicate an independent evolution of organ-specific traits [21, 70], albeit linked by GD to A_mass_ and Rd_mass_ (Fig. 6B), likely driven by hydraulics and physiological metabolic processes [71, 72]. For example, leaf photosynthesis and respiration metabolism are related to the acquisition and dissipation of carbon and GD determines the ability of the above-ground bamboo system, particularly leaves, to extract mineral elements (e.g. N and P) and water from the soil for transport to leaf blades through culm sieve tubes and conduits [73, 74] that, in turn, transport fixed carbon belowground for the construction of rhizomes [23, 75]. Due to the syndrome of woody bamboo, we focused on the morphological traits of culms, and the physiological metabolic and hydrodynamic processes of culms and leaves still need to be tested with more relevant functional traits. In addition, coordinated functional associations of culm and leaf traits (morphology, nutrient and water transport and biomass allocation ratio) in individual plants are usually also regulated by light resources [76] and soil nutrient availability levels and ratios (especially N and P) [77–79]. Therefore, biological processes that require multi-organ synergies link leaf and culm functional traits in individual plants.

Leaf and culm are the main functional modules of carbon acquisition and material transport in plants [80], and the correlation of functional traits indicates the coupling of physiological processes between them [71, 81], for example, GD (with a betweenness coefficient of 5) links leaves and culms of woody bamboo through hydraulics and physiological-metabolic processes. In addition, we found that leaf N has the highest betweenness (5) in woody bamboo leaves (Fig. 6B), suggesting that N acts as a ‘bridge’ trait, linking the A_mass_, Rd_mass_ and P, which is consistent with previous studies [82]. This result can be interpreted as follows: on the one hand, N participates in physiological processes such as photosynthesis in the form of active compounds such as Rubisco enzyme [66, 67]; on the other hand, it also maintains nutrient stoichiometric stability in leaves together with P [83] and regulates the balance between physiological-metabolic processes and nutrient concentration in leaves to a certain extent [84, 85]. Besides, photosynthetic and respiratory rates, which require the participation of N, are also limited by LMA, which is constructed and thus determined from photosynthetic products [86]. This explains that A_mass_ and Rd_mass_ have the second highest betweenness coefficients in woody bamboo leaves.

## Conclusion

We observed extensive interspecific variations among 77 woody bamboo species in the common gardens, dominated by heritable legacy and the climate of the occurrence sites, consistent with the principles of biogeochemical niches. We confirmed the woody bamboo leaf economic spectrum clustered at the acquisition end of the global leaf economics spectrum and found differences in leaf functional trait trade-offs between this taxon and non-bamboo species resulting from the woody bamboo syndromes, as manifested by low leaf construction costs, high nutrient use efficiency, and rapid physiological processes. Leaf elemental composition was associated with morphophysiology traits in woody bamboo leaf functional traits; A_mass_ and Rd_mass_ were leaf trait hubs, linking leaf traits to culm traits through GD. In summary, our findings provide a foundation for understanding trade-off strategies and ecological adaptations in woody bamboos, expanding the taxonomic units considered for global plant functional trait studies.

Given the current scarcity of functional trait studies on woody bamboos worldwide, we tested functional trait variations and trade-offs in this taxon in a common garden. Although this is one of the largest datasets of functional traits for woody bamboo leaves and culms available for the number of species to date, there is still a need to test at the woody bamboo habitat occurrence sites and incorporate as many functional traits and environmental variables as possible to reveal the ecological adaptive strategies of woody bamboos comprehensively.

### Electronic supplementary material

Below is the link to the electronic supplementary material.

Supplementary Material 1

## Data Availability

The datasets used and/or analyzed during the current study are available from the corresponding author on reasonable request.
