# Supplementary material for: Variations and trade-offs in leaf and culm functional traits among 77 woody bamboo species
Source: BMC Plant Biol. 2024 May 10;24:387. doi: 10.1186/s12870-024-05108-2 (PMC11084126; doi:10.1186/s12870-024-05108-2)
Supplement: Supplementary file 1 — Supplementary Material 1 [file 12870_2024_5108_MOESM1_ESM.docx]

# Supplementary Figures and Tables

## Table S1 Woody bamboo species, introduction country, distribution of origin and growth form.

| Code | Tribe | Genus and  species | Introduction  country | Distribution of origin | Growth  form |
| --- | --- | --- | --- | --- | --- |
| 1 | Arundinarieae | *Indocalamus*  *hirtivaginatus* | China | Asia-temperate: China. | Shrubby |
| 2 | Arundinarieae | *Indocalamus*  *herklotsii* | China | Asia-temperate: China. | Shrubby |
| 3 | Arundinarieae | *Shibataea*  *kumasasa* | China | Asia-temperate: Caucasus, China, and eastern Asia. | Shrubby |
| 4 | Arundinarieae | *Semiarundinaria*  *densiflora* | China | Asia-temperate: China. | Shrubby |
| 5 | Arundinarieae | *Indocalamus*  *guangdongensis* | China | Asia-temperate: China. | Shrubby |
| 6 | Arundinarieae | *Oligostachyum*  *oedogonatum* | China | Asia-temperate: China. | Shrubby |
| 7 | Arundinarieae | *Pleioblastus*  *simonii* | China | Asia-temperate: Caucasus and eastern Asia. Asia-tropical: India. | Arborescent |
| 8 | Arundinarieae | *Phyllostachys*  *propinqua* | China | Asia-temperate: China. | Arborescent |
| 9 | Arundinarieae | *Phyllostachys*  *edulis* | China | Asia-temperate: Caucasus, China, and eastern Asia. Asia-tropical: Indo-China. | Arborescent |
| 10 | Arundinarieae | *Pleioblastus*  *argenteostriatus* | China | Asia-temperate: Caucasus, China, and eastern Asia. Pacific: northwestern. | Shrubby |
| 11 | Arundinarieae | *Phyllostachys*  *robustiramea* | China | Asia-temperate: China. | Arborescent |
| 12 | Arundinarieae | *Phyllostachys*  *bambusoides* | China | Asia-temperate: China. | Arborescent |
| 13 | Arundinarieae | *Pleioblastus*  *maculatus* | China | Asia-temperate: China. | Shrubby |
| 14 | Arundinarieae | *Pseudosasa*  *amabilis* | China | Asia-temperate: China. Asia-tropical: Indo-China. | Shrubby |
| 15 | Arundinarieae | *Pseudosasa*  *subsolida* | China | Asia-temperate: China. | Shrubby |
| 16 | Arundinarieae | *Arundinaria*  *fargesii* | China | Asia-temperate: China. | Shrubby |
| 17 | Arundinarieae | *Indosasa*  *crassiflora* | China | Asia-temperate: China. Asia-tropical: Indo-China. | Shrubby |
| 18 | Arundinarieae | *Indosasa*  *longispicata* | China | Asia-temperate: China. | Arborescent |
| 19 | Arundinarieae | *Indosasa*  *sinica* | China | Asia-temperate: China. | Arborescent |
| 20 | Arundinarieae | *Indosasa*  *glabrata* | China | Asia-temperate: China. | Arborescent |
| 21 | Arundinarieae | *Indosasa*  *patens* | China | Asia-temperate: China. | Arborescent |
| 22 | Arundinarieae | *Pseudosasa*  *disticha* | Japan | Asia-temperate: China, Japan. | Shrubby |
| 23 | Arundinarieae | *Chimonobambusa*  *communis* | China | Asia-temperate: China. | Shrubby |
| 24 | Arundinarieae | *Chimonobambusa*  *tumidissinoda* | Japan | Asia-temperate: China, Japan. | Shrubby |
| 25 | Arundinarieae | *Chimonobambusa*  *purpurea* | China | Asia-temperate: China. | Shrubby |
| 26 | Arundinarieae | *Chimonobambusa*  *quadrangularis* | China | Asia-temperate: Caucasus, China, and eastern Asia. Asia-tropical: Indo-China. Australasia: New Zealand. | Shrubby |
| 27 | Arundinarieae | *Phyllostachys*  *heteroclada* | China | Asia-temperate: China. | Arborescent |
| 28 | Arundinarieae | *Phyllostachys*  *fimbriligula* | China | Asia-temperate: China. | Arborescent |
| 29 | Arundinarieae | *Phyllostachys*  *vivax* | China | Asia-temperate: China. | Arborescent |
| 30 | Arundinarieae | *Semiarundinaria*  *fastuosa* | Japan | Asia-temperate: Caucasus, China, and eastern Asia. Australasia: New Zealand. | Shrubby |
| 31 | Arundinarieae | *Indosasa*  *shibataeaoides* | China | Asia-temperate: China. | Arborescent |
| 32 | Arundinarieae | *Phyllostachys*  *stimulosa* | China | Asia-temperate: China. | Arborescent |
| 33 | Arundinarieae | *Phyllostachys*  *violascens* | China | Asia-temperate: China. | Arborescent |
| 34 | Arundinarieae | *Phyllostachys*  *sulphurea* | China | Asia-temperate: Caucasus and China. | Arborescent |
| 35 | Arundinarieae | *Pleioblastus*  *amarus* | China | Asia-temperate: China. | Arborescent |
| 36 | Arundinarieae | *Sinobambusa*  *tootsik* | Japan | Asia-temperate: China and eastern Asia. | Arborescent |
| 37 | Arundinarieae | *Acidosasa*  *notata* | China | Asia-temperate: China. | Shrubby |
| 38 | Arundinarieae | *Indocalamus*  *bashanensis* | China | Asia-temperate: China. | Shrubby |
| 39 | Bambuseae | *Bambusa*  *subaequalis* | Japan | Asia-temperate: China and eastern Asia. | Arborescent |
| 40 | Arundinarieae | *Pleioblastus*  *viridistriatus* | Japan | Asia-temperate: Caucasus and eastern Asia. Australasia: New Zealand. | Shrubby |
| 41 | Arundinarieae | *Sasa*  *masamuneana* | China | Asia-temperate: eastern Asia. | Shrubby |
| 42 | Arundinarieae | *Semiarundinaria*  *sinica* | China | Asia-temperate: China. | Shrubby |
| 43 | Arundinarieae | *Pleioblastus*  *gramineus* | Japan | Asia-temperate: eastern Asia. Australasia: New Zealand. | Shrubby |
| 44 | Arundinarieae | *Acidosasa*  *venusta* | China | Asia-temperate: China. | Shrubby |
| 45 | Arundinarieae | *Phyllostachys*  *makinoi* | China | Asia-temperate: China and eastern Asia. | Arborescent |
| 46 | Arundinarieae | *Pseudosasa*  *japonica* | Japan | Africa: Macaronesia. Asia-temperate: Caucasus, western Asia, China, and eastern Asia. Asia-tropical: Indo-China. Australasia: New Zealand. | Shrubby |
| 47 | Arundinarieae | *Indocalamus*  *longiauritus* | China | Asia-temperate: China. | Shrubby |
| 48 | Arundinarieae | *Oligostachyum*  *spongiosum* | China | Asia-temperate: China. | Arborescent |
| 49 | Arundinarieae | *Oligostachyum*  *scabriflorum* | China | Asia-temperate: China. | Shrubby |
| 50 | Arundinarieae | *Phyllostachys*  *nidularia* | China | Asia-temperate: China. | Arborescent |
| 51 | Arundinarieae | *Indocalamus*  *hunanensis* | China | Asia-temperate: China. | Shrubby |
| 52 | Arundinarieae | *Drepanostachyum*  *stoloniforme* | China | Asia-temperate: China. | Lianas |
| 53 | Bambuseae | *Bambusa*  *tulda* | India | Asia-temperate: China. Asia-tropical: India and Indo-China. Pacific: northwestern. | Arborescent |
| 54 | Bambuseae | *Dendrocalamus*  *strictus* | India | Africa: western Indian ocean. Asia-temperate: eastern Asia. Asia-tropical: India, Indo-China, and Malesia. Pacific: northwestern. | Arborescent |
| 55 | Bambuseae | *Gigantochloa*  *macrostachya* | Thailand | Asia-tropical: India and Indo-China. | Arborescent |
| 56 | Bambuseae | *Thyrsostachys*  *oliveri* | Thailand | Asia-temperate: China. Asia-tropical: India and Indo-China. | Arborescent |
| 57 | Bambuseae | *Gigantochloa*  *verticillata* | India | Asia-temperate: China. Asia-tropical: Indo-China and Malesia. | Arborescent |
| 58 | Bambuseae | *Bambusa*  *bambos* | Indonesia | Africa: western Indian ocean. Asia-tropical: India, Indo-China, and Malesia. South America: Mesoamericana and northern South America. | Arborescent |
| 59 | Bambuseae | *Dendrocalamus*  *semiscandens* | China | Asia-temperate: China. | Arborescent |
| 60 | Bambuseae | *Dendrocalamus*  *sericeus* | India | Asia-tropical: Indo-China. | Arborescent |
| 61 | Bambuseae | *Schizostachyum*  *diffusum* | China | Asia-temperate: China. | Arborescent |
| 62 | Bambuseae | *Thyrsostachys*  *siamensis* | Thailand | Asia-temperate: China. Asia-tropical: India and Indo-China. | Arborescent |
| 63 | Arundinarieae | *Fargesia*  *papyrifera* | China | Asia-temperate: China. | Shrubby |
| 64 | Bambuseae | *Dendrocalamus*  *yunnanicus* | China | Asia-temperate: China. | Arborescent |
| 65 | Bambuseae | *Dendrocalamus*  *fugongensis* | China | Asia-temperate: China. | Arborescent |
| 66 | Bambuseae | *Schizostachyum*  *funghomii* | China | Asia-temperate: China. | Arborescent |
| 67 | Bambuseae | *Bambusa*  *chungii* | China | Asia-temperate: China. Asia-tropical: Indo-China. | Arborescent |
| 68 | Bambuseae | *Dendrocalamus*  *sinicus* | China | Asia-temperate: China. | Arborescent |
| 69 | Arundinarieae | *Fargesia*  *stenoclada* | China | Asia-temperate: China. | Shrubby |
| 70 | Bambuseae | *Dendrocalamus*  *giganteus* | China | Africa: western Indian ocean. Asia-temperate: China and eastern Asia. Asia-tropical: India, Indo-China, and Malesia. | Arborescent |
| 71 | Bambuseae | *Dendrocalamus*  *membranaceus* | China | Asia-temperate: China. Asia-tropical: India and Indo-China. | Arborescent |
| 72 | Bambuseae | *Schizostachyum*  *brachycladum* | Malaysia | Asia-tropical: Indo-China and Malesia. | Arborescent |
| 73 | Bambuseae | *Dendrocalamus*  *asper* | Malaysia | Asia-temperate: China and eastern Asia. Asia-tropical: India, Indo-China, and Malesia. | Arborescent |
| 74 | Bambuseae | *Bambusa*  *lako* | Malaysia | Asia-tropical: Malesia. | Arborescent |
| 75 | Bambuseae | *Cephalostachyum*  *pergracile* | India | Asia-temperate: China. Asia-tropical: India and Indo-China. | Arborescent |
| 76 | Bambuseae | *Bambusa*  *beecheyana* | China | Asia-temperate: China and eastern Asia. Asia-tropical: Indo-China. | Arborescent |
| 77 | Bambuseae | *Gigantochloa*  *pruriens* | Malesia | Asia-tropical: Malesia. | Arborescent |

## Table S2 Results from Bayesian phylogenetic linear mixed models with temperature and precipitation variables taking into account the phylogeny importance (n = 54).

| Dep. | Bayesian model |  |  |  |  |  |  |
| --- | --- | --- | --- | --- | --- | --- | --- |
| Var | Model | Fixed variables statistics | | | | | Model statistics |
| GD | Log (GD) ~ MAT + MAP |  | post.mean | l-95%CI | u-95%CI | eff.samp | R^2^_c_ = 0.7644 |
|  | + (random = phylogeny +species) | (Intercept) | -0.7392 | -2.321 | 0.8274 | 800 | R^2^_m_ = 0.0731 |
|  |  | MAT | 0.04408 | -0.03024 | 0.1246 | 800 | R^2^_p_ = 0.469 |
|  |  | MAP | -0.00003158 | -0.0003948 | 0.0003143 | 952 | R^2^_s_ = 0.2222 |
| ITL | Log (ITL) ~ MAT + MAP |  | post.mean | l-95%CI | u-95%CI | eff.samp | R^2^_c_ = 0.7092 |
|  | + (random = phylogeny +species) | (Intercept) | -1.3460164 | -3.1027628 | 0.5261315 | 557 | R^2^_m_ = 0.1669 |
|  |  | MAT | 0.0905821 | 0.0034328 | 0.1780596 | 454 | R^2^_p_ = 0.2212 |
|  |  | MAP | -0.0001322 | -0.000526 | 0.0002429 | 800 | R^2^_s_ = 0.3211 |
| LMA | Log (LMA) ~ MAT + MAP |  | post.mean | l-95%CI | u-95%CI | eff.samp | R^2^_c_ = 0.551 |
|  | + (random = phylogeny +species) | (Intercept) | 0.71439 | -0.8552464 | 2.4819132 | 623 | R^2^_m_ = 0.0751 |
|  |  | MAT | -0.0504064 | -0.1193338 | 0.0415251 | 656 | R^2^_p_ = 0.1118 |
|  |  | MAP | 0.000117 | -0.0003719 | 0.0005692 | 803 | R^2^_s_ = 0.3641 |
| N | Log (N) ~ MAT + MAP |  | post.mean | l-95%CI | u-95%CI | eff.samp | R^2^_c_ = 0.6608 |
|  | + (random = phylogeny +species) | (Intercept) | -0.8073 | -2.624 | 0.9236 | 526 | R^2^_m_ = 0.0851 |
|  |  | MAT | 0.04761 | -0.03894 | 0.1315 | 620 | R^2^_p_ = 0.2955 |
|  |  | MAP | 0.00002822 | -0.0003661 | 0.000419 | 800 | R^2^_s_ = 0.2802 |
| P | Log (P) ~ MAT + MAP |  | post.mean | l-95%CI | u-95%CI | eff.samp | R^2^_c_ = 0.5622 |
|  | + (random = phylogeny +species) | (Intercept) | -0.5087 | -2.358 | 1.618 | 300 | R^2^_m_ = 0.0675 |
|  |  | MAT | 0.02653 | -0.08177 | 0.1043 | 359 | R^2^_p_ = 0.1584 |
|  |  | MAP | 0.00008779 | -0.000385 | 0.0005525 | 800 | R^2^_s_ = 0.3363 |
| A_area_ | Log (A_area_) ~ MAT + MAP |  | post.mean | l-95%CI | u-95%CI | eff.samp | R^2^_c_ = 0.8743 |
|  | + (random = phylogeny +species) | (Intercept) | -1.0885874 | -2.7976051 | 0.6220233 | 800 | R^2^_m_ = 0.1133 |
|  |  | MAT | 0.0465225 | -0.0350309 | 0.125321 | 800 | R^2^_p_ = 0.6238 |
|  |  | MAP | 0.0002477 | -0.0001187 | 0.0005409 | 800 | R^2^_s_ = 0.1372 |
| Rd_area_ | Log (Rd_area_) ~ MAT + MAP |  | post.mean | l-95%CI | u-95%CI | eff.samp | R^2^_c_ = 0.9089 |
|  | + (random = phylogeny +species) | (Intercept) | -1.306 | -2.933 | 0.2908 | 800 | R^2^_m_ = 0.1633 |
|  |  | MAT | 0.07223 | 0.003269 | 0.1408 | 983 | R^2^_p_ = 0.6102 |
|  |  | MAP | 0.0001547 | -0.00009671 | 0.0004562 | 800 | R^2^_s_ = 0.1354 |

MAT: mean annual temperature; MAP: mean annual precipitation. R^2^_c_= Percentage of var. explained by all the model (fixed + random). R^2^_m_= Percentage of var. explained by fixed factors. R^2^_p_ = Percentage of var. explained by phylogeny. R^2^_s_=Percentage of variance explained species.

## Table S3 Results of Wilks’ Lambda test in canonical correlation analysis (n = 77).

| Root scores | stat | approx | df1 | df2 | p.value |
| --- | --- | --- | --- | --- | --- |
| 1 to 2 | 0.670 | 5.315 | 6 | 144 | < 0.001 |
| 2 to 2 | 0.993 | 0.241 | 2 | 73 | 0.786 |


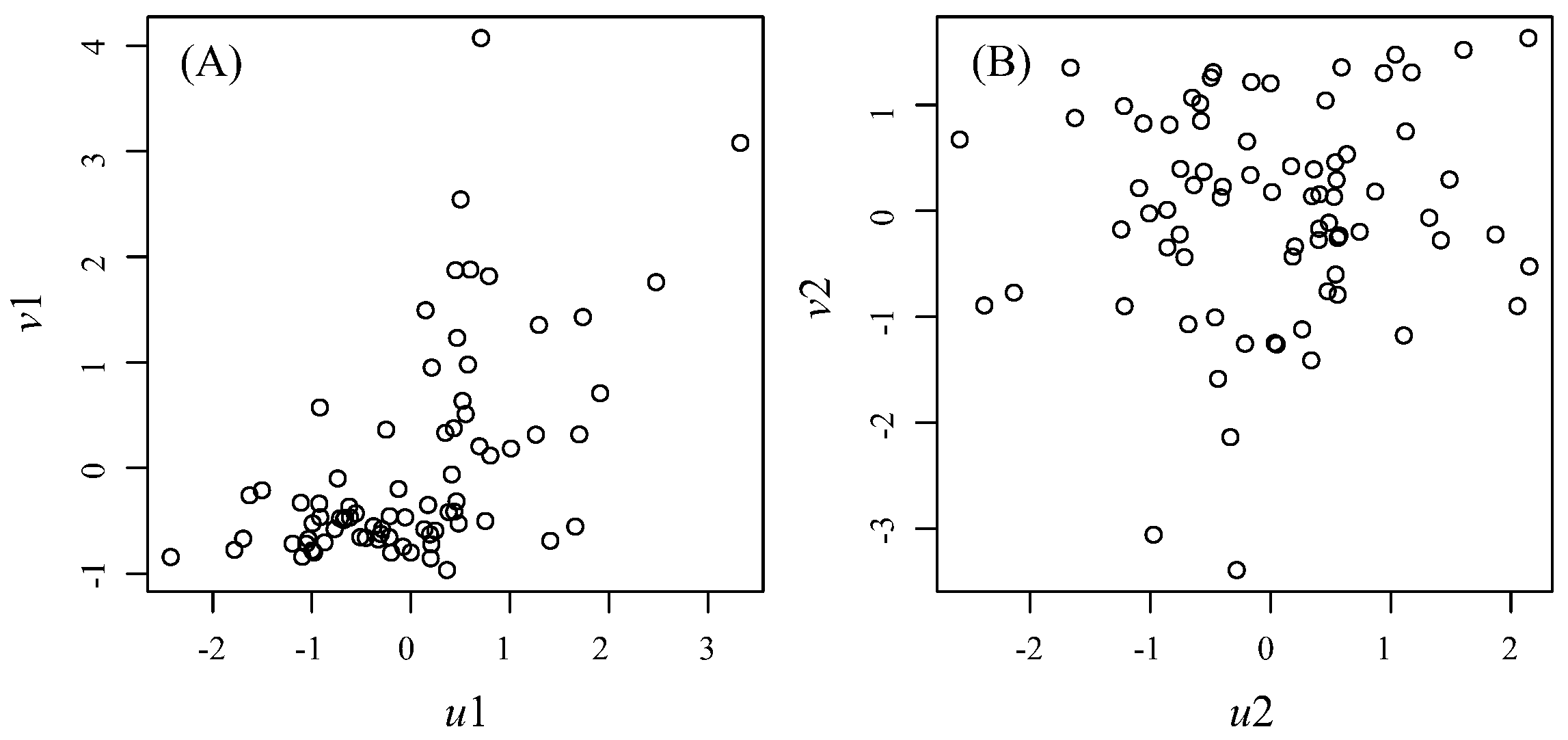


## Fig. S1 The correlations of root scores 1 (A) and 2 (B) for the leaf elementome (*v*) and the morphophysiology traits (*u*) in canonical correlation analysis (n = 77).
